# Co-incubation of Bacillus spp. alleviated the growth and gas production of potentially harmful bacteria in different in vitro methodologies

**DOI:** 10.3168/jdsc.2025-0896

**Published:** 2026-05-22

**Authors:** Lena Capern, Nina Milora, Emily George, Audrey Segura, Raphaele Gresse, Oscar Queiroz, Giuseppe Copani, Bruno I. Cappellozza

**Affiliations:** 1 Novonesis, Hørsholm 2970, Denmark; 2 Novonesis, Franklinton, NC 27525; 3 Novonesis, Lyngby 2800, Denmark

## Abstract

A series of experiments evaluated the effects of a *Bacillus*-based direct-fed microbial (DFM) on the growth of potentially harmful bacteria. Experiment 1 evaluated the effects of co-incubating *Bacillus licheniformis* 809 or *Bacillus subtilis* 810 with *C. perfringens* type A on in vitro gas production and *Clostridium* counts. This methodology was performed using the in vitro gas production Ankom RF system bottle. Bottles were incubated with *C. perfringens* type A (CPF) or with the addition of individual DFM strains (*B. licheniformis* 809 [BL] or *B. subtilis* 810 [BS]). Gas production of the different treatments was recorded for 24 h, and then enumeration of the strains was performed. A treatment × hour interaction was observed, as CPF grew faster than either BL or BS from 7 to 24 h, whereas BS grew faster than BL at 7 h. Adding BL or BS reduced gas production versus CPF from 18 to 24 h (treatment × hour interaction). Moreover, the co-incubation of BL or BS with *C. perfringens* type A significantly reduced the counts of these potentially harmful bacteria by 63% and 69%, respectively. Experiment 2 was performed by using an agar-diffusion assay to evaluate *C. perfringens* types A, B, C, D, and E; *Fusobacterium necrophorum*; *Trueperella pyogenes*; and *L. monocytogenes*. After incubation, the zone of decreased growth was measured. The *Bacillus*-based DFM addition resulted in efficacy against *C. perfringens* that ranged from 10.5 mm (type C) to 15.5 mm (types A and E). Moreover, for *T. pyogenes* and *F. necrophorum*, the clearance zone was in the order of 11.5 and 8.7 mm, respectively. Three strains of *L. monocytogenes* were assessed following 4 h of incubation with BL and BS, and zones between 10.5 and 11.5 mm were reported. In summary, this series of in vitro experiments demonstrated the inhibitory activity of BL and BS against selected potentially harmful bacteria of interest for livestock.

## Highlights

• *Clostridium perfringens* type A outgrew *Bacillus* spp.
• Reduced counts of *C. perfringens* were observed.
• *Bacillus* spp. alleviated the growth of potentially harmful bacteria.
• These positive effects were observed in gram-positive and gram-negative bacteria.

## Graphical Abstract

**Figure:**
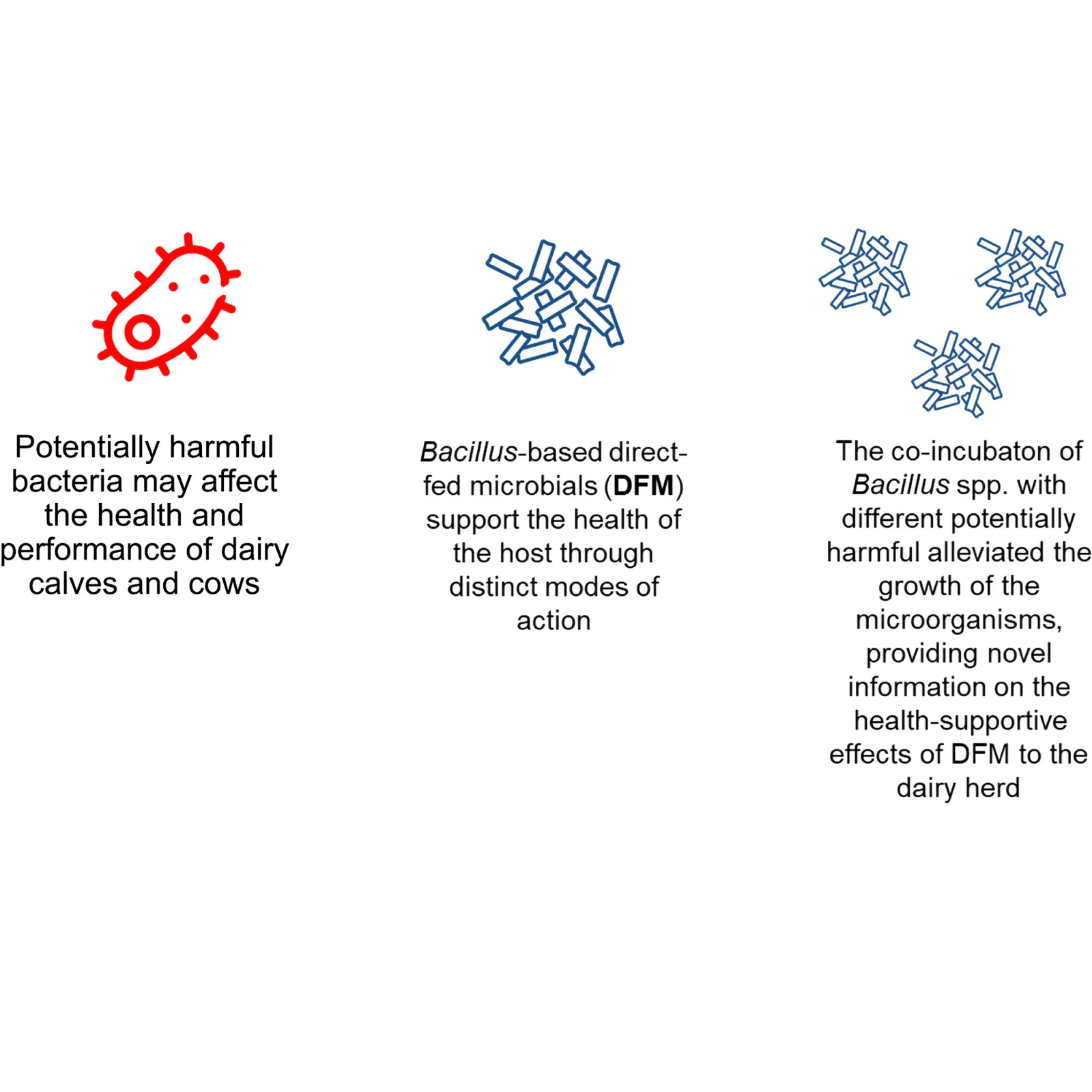
**Summary:** Potentially harmful bacteria are known to affect the health and performance of dairy cows and calves, whereas *Bacillus*-based direct-fed microbials have been shown to support the health and performance of the dairy cattle herd via distinct modes of action. We have demonstrated, under different in vitro methodologies, that *Bacillus licheniformis* 809 and *B. subtilis* 810 alleviate the growth of a different set of potentially harmful bacteria, ranging from gram-negative to gram-positive microorganisms. These results were noticeable even when the potentially harmful bacteria outgrew *B. licheniformis* 809 and *B. subtilis* 810. These results help us to elucidate the current data available in the literature demonstrating the positive effects of *Bacillus* spp. on the health and performance of the dairy cattle herd.

Dairy calves and cows are often exposed to several stressors during their productive lives that can make them more susceptible to potentially adverse health events and ultimately affect their short-, medium- and long-term performance in the herd. As an example, calves presenting diarrhea during the preweaning period gained less BW (Soberon et al., 2012), produced less milk (Heinrichs and Heinrichs, 2011), and had a greater likelihood of being culled in the first 300 DIM (Goh et al., 2024). Likewise, periparturient dairy cows showing ≥1 episodes of clinical adverse health events, such as metritis, had a lower peak milk yield and daily milk yield when compared with healthy cows (Carvalho et al., 2019). Although the root cause of these adverse health events may be unknown, potentially harmful bacteria, including *Clostridium perfringens*, *Fusobacterium necrophorum*, and *Trueperella pyogenes*, may play a key role in the occurrence of these events (Uzal et al., 2014; Cunha et al., 2018; Tamai et al., 2022).

Bacteria-based directed microbials (**DFM**) support the health of the host through diverse modes of action, when offered under the recommended amounts (FAO/WHO, 2001). Although different species of bacteria may be fed to ruminants (Cappellozza et al., 2025), recent publications have highlighted the health-supportive effects of *Bacillus* spp. for dairy calves (Lucey et al., 2021; Magalhães et al., 2024) and cows (Goetz et al., 2023, 2024). These positive health-related results could be due to the direct effects against some potentially harmful bacteria (Luise et al., 2022). However, given the complexity and the diversity of microorganisms that could be involved in these events, it is paramount to understand the effects of DFM, and more specifically *Bacillus*-based DFM, on the growth of potentially harmful bacteria of importance to the dairy operations, such as *C. perfringens* (gut health), *F. necrophorum*, *T. pyogenes* (rumen and reproductive tract health), and *Listeria monocytogenes* (gut health). Hence, we hypothesized that *Bacillus* spp. would reduce the growth of *C. perfringens*, *F. necrophorum*, *T. pyogenes*, and *L. monocytogenes*. Therefore, this manuscript evaluated the effects of adding *B. licheniformis* 809 (**BL**) and *B. subtilis* 810 (**BS**) on the growth of the aforementioned potentially harmful bacteria.

In experiment 1, a gas production assay was used as a way to evaluate the growth of the bacteria in an anaerobic environment, as gas production is an indication of bacteria growth. In brief, 90 mL of brain heart infusion (**BHI**) was added in 100 mL blue cap bottle and autoclaved for 15 min at 121°C. Bottles were flushed with carbon dioxide (CO_2_) and incubated anaerobically overnight before running the experiment. Then, *C. perfringens* type A (CHCC #14327; **CPF**) or CPF with 0.5 mL of the individual DFM strains (*B. licheniformis* 809 [CPF+BL] or *B. subtilis* 810 [CPF+BS]) were incubated. The *C. perfringens* type A was grown overnight on a blood plate at 37°C, and a specific colony was inoculated in BHI broth for up to 2 d for further inoculation in the Ankom RF bottles. *Bacillus* spp. were grown overnight on a trypticase soy agar (**TSA**) plate, and one colony of each strain was inoculated in BHI broth, grown overnight, and added in the bottles at the dosage of 1.0 × 10^6^ cfu/mL. The bottles were then flushed with CO_2_ before the Ankom RF module was screwed on. The bottles were incubated at 37°C, gas production was recorded (in psi) hourly for 24 h, and then enumeration of the strains was performed by visually identifying the morphology of clostridia and bacilli. For both gas production and bacteria count endpoints, samples were analyzed in independent triplicates.

All data were analyzed with PROC MIXED of SAS (version 9.4, SAS Institute Inc., Cary, NC), considering bottle as the experimental unit. Gas production was analyzed over time, and therefore treatment × hour interactions were also tested using hour as a repeated factor, and the autoregressive covariance structure was based on the lowest Akaike information criterion. Last, the counts of *C. perfringens* type A at the end of the 24-h period were analyzed using treatment as the fixed factor. Significance was set at *P* ≤ 0.05, and results were presented according to the highest-order interaction.

In experiment 2, an agar-diffusion assay was performed by evaluating the *C. perfringens* type A (CHCC #14327), type B (native strain), type C (CHCC #18121), type D (native strain), and type E (CHCC #19492); *F. necrophorum* (CHCC #28517); *T. pyogenes* (CHCC #48578); and *L. monocytogenes* (ATCC #19111, 19114, and baa-3153). For all bacterial pathogens evaluated, samples were analyzed in independent triplicates (n = 3). After coincubation with DFM added to the agar plate, the zone of decreased growth for the potential pathogen was measured from full growth to full growth, with a lower limit of 3.5 mm. In all plates, wells containing only the media were used as the controls for the subsequent assessments.

*Clostridium perfringens* was assessed following the same methodology previously described (Guimaraes et al., 2023). Briefly, on d 1, *C. perfringens* serotypes were inoculated on TSA with sheep blood (**TSA-SB**) medium and incubated at 37°C anaerobically for 18 h. On the same day, independent cultures in BHI broth of DFM (*B. licheniformis* 809 and *B. subtilis* 810; Bovacillus, Novonesis, Lyngby, Denmark) were prepared and incubated aerobically overnight at 37°C. On d 2, Perfringens Agar Base (Oxoid) was prepared and cooled to 50°C, whereas the overnight cultures of *C. perfringens* serotypes were suspended by using a cotton swab in a maximum recovery diluent (**MRD**) medium to a density of 1.3 McFarland. Following this step, 35 mL of melted agar and 10 μL of pathogen suspension were mixed in a 50-mL Falcon tube (Corning Life Sciences, Corning, NY), and the mixture was cast in Omnitray plates with an immediate application of the Nunc Immuno TSP (Thermo Fisher Scientific, Waltham, MA). The plates were then left to solidify for 20 min before removing the lid. Thereafter, plates were allowed to dry with lid on for an additional 20 min. Last, the overnight DFM strain cultures were mixed (final pH = 6.5) and 10 µL were applied to selected wells in the agar. The plates were incubated anaerobically at 42°C for 24 h until clear growth of *C. perfringens* were observed, after which the plates were immediately read.

For assessment of *Fusobacterium necrophorum* and *Trueperella pyogenes*, on d 1, both were inoculated on TSA-SB medium and incubated at 37°C anaerobically for 48 h. On d 2, cultures in BHI broth of the DFM (BL and BS) were prepared and incubated aerobically overnight at 37°C. On d 3, Wilkins Chalgren Anaerobe Agar (Oxoid) was prepared and cooled to 50°C, and the cultures of *F. necrophorum* and *T. pyogenes* were suspended by using a cotton swab in a MRD medium to a density of 0.5 McFarland. Following this step, 35-mL melted agar and 10 μL of pathogen suspension were mixed in a 50-mL Falcon tube, and the mixture was cast in Omnitray plates with an immediate application of the lid. The plates were then left to solidify for 20 min before the lid was removed. Thereafter, plates were allowed to dry with a normal lid on for an additional 30 min, and then 10 µL of the DFM overnight culture was applied to selected wells in the agar (pH = 7.0). The plates were incubated anaerobically at 37°C for 48 h, after which the plates were read.

For assessment of *Listeria monocytogenes*, on d 1, the strains were streaked from freezer stocks into BHI agar plates for isolated colonies, and the plates were incubated overnight at 35°C. The following day, the plates were checked for purity and for the presence of the isolated colonies. A single colony of each *L. monocytogenes* strain was then inoculated in 5 mL of sterile BHI, and the cultures were incubated aerobically at 35°C overnight with shaking at 200 rpm. On d 3, 0.25 g of the commercial product containing BL and BS was added into individual sterile 15-mL glass test tubes. Then, 5 mL of sterile TSA was added to each tube, the tubes were vortexed until an even suspension was observed, and an aerobic incubation was performed for 4 h at 35°C with shaking at 200 rpm. On the same day, *L. monocytogenes* strains were checked for vegetative growth, cultures were washed and then centrifuged at 10,000 × *g* for 30 s at 35°C, and the cell pellet was mixed with MRD and vortexed until the pellet was fully resuspended into solution. The optical density normalization of the *L. monocytogenes* strains was performed by adding 5 mL of sterile MRD and 100 µL of washed *Listeria* culture to a 15-mL glass tube, and the mixture was then vortexed until homogenized. The turbidity of the culture was evaluated using a densiometer, and the washed cultures were added to a MRD tube until a final turbidity of 0.5 MacFarland was reached (pH = 6.0). The incubation of the plates was performed anaerobically at 35°C for 24 to 48 h, whereas readings were performed at 4 h.

A treatment × hour interaction was observed on the growth curves of CPF, BL, and BS (*P* < 0.001), as CPF grew faster than either BL or BS from 7 to 24 h (*P* < 0.01), whereas BS grew faster than BL at 7 h (*P* = 0.03), and no differences were observed at 24 h (*P* = 0.88; Figure 1A). A similar treatment × hour interaction was observed on gas production (*P* < 0.001), as adding BL or BS reduced gas production versus CPF alone from 18 to 24 h (*P* ≤ 0.05). Moreover, CPF+BL had a lower gas production versus CPF+BS at 23 and 24 h (*P* ≤ 0.04; Figure 1B). Last, the co-incubation of BL and BS with *C. perfringens* type A significantly reduced (*P* < 0.01) its counts at 24 h by 63% and 69%, respectively (Figure 1C).

**Figure 1:**
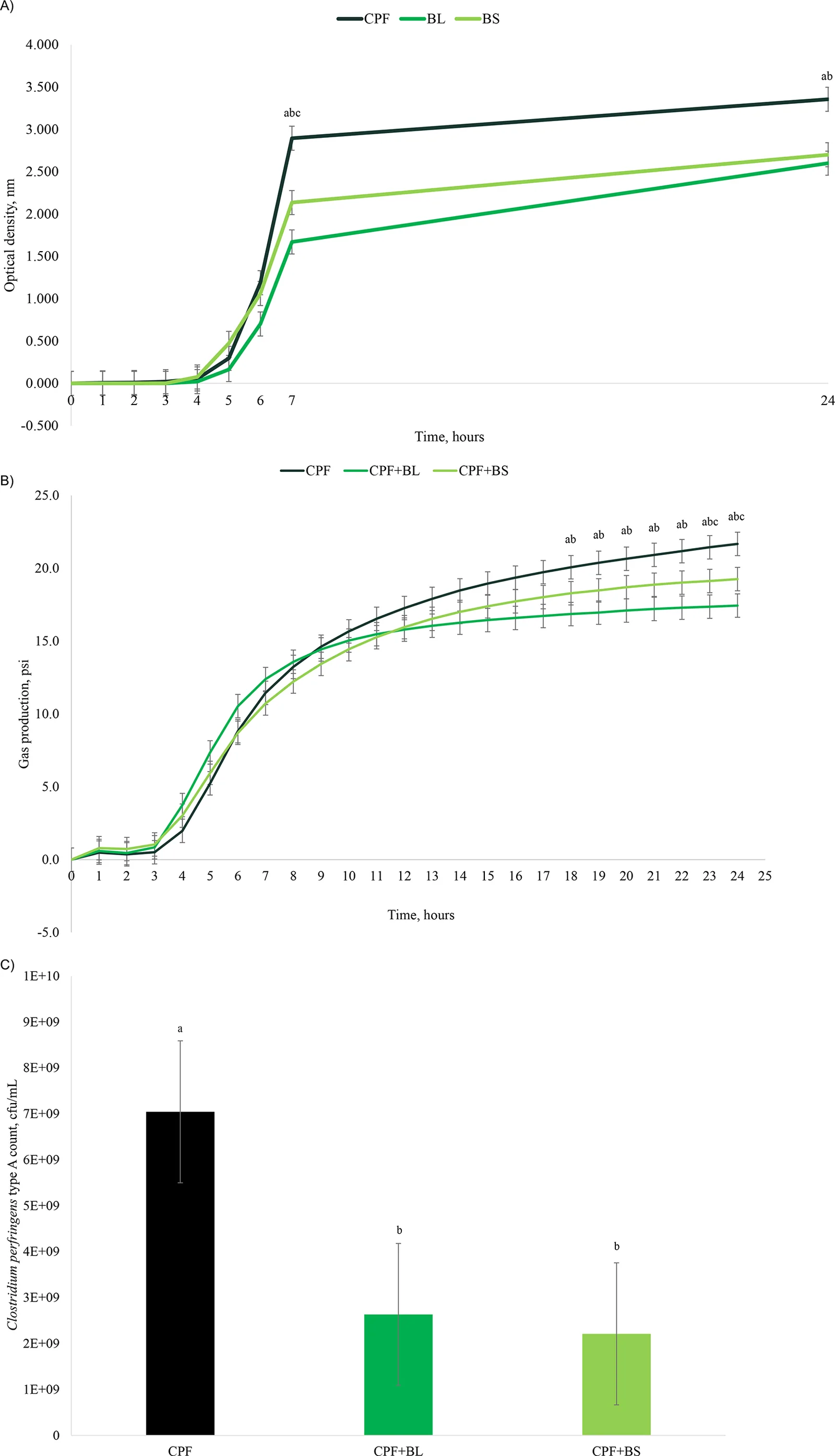
(A) Growth curves of *Clostridium perfringens* type A (CPF), *B. licheniformis* 809 (BL), and *B. subtilis* 810 (BS) in brain heart infusion (BHI) medium for 24 h. (B) In vitro gas production curve of *Clostridium perfringens* type A incubated alone (CPF) or with the addition of *Bacillus licheniformis* 809 (CPF+BL) or *B. subtilis* 810 (CPF+BS) for 24 h. A treatment × hour interaction was observed (*P* < 0.0001). (C) Counts of *Clostridium perfringens* type A (cfu/g) when incubated alone (CPF) or with the addition of *Bacillus licheniformis* 809 (CPF+BL) or *B. subtilis* 810 (CPF+BS) for 24 h. Different lowercase letters denote significant differences at *P* ≤ 0.05. Error bars are SEM.

Supporting the results from experiment 1, *Bacillus*-based DFM addition was efficacious against *C. perfringens*, yielding clearance zones that ranged from 10.5 mm (type C) to 15.5 mm (types A and E; Figure 2A). Moreover, for *T. pyogenes* and *F. necrophorum*, the clearance zone was in the order of 11.5 and 8.7 mm, respectively (Figure 2B). Last, co-incubating *Bacillus* spp. yielded clearance zones of 11.1, 11.5, and 10.5 mm when the 19111, 19114, and baa-3153 strains of *L. monocytogenes* were evaluated, respectively (Figure 2C).

**Figure 2:**
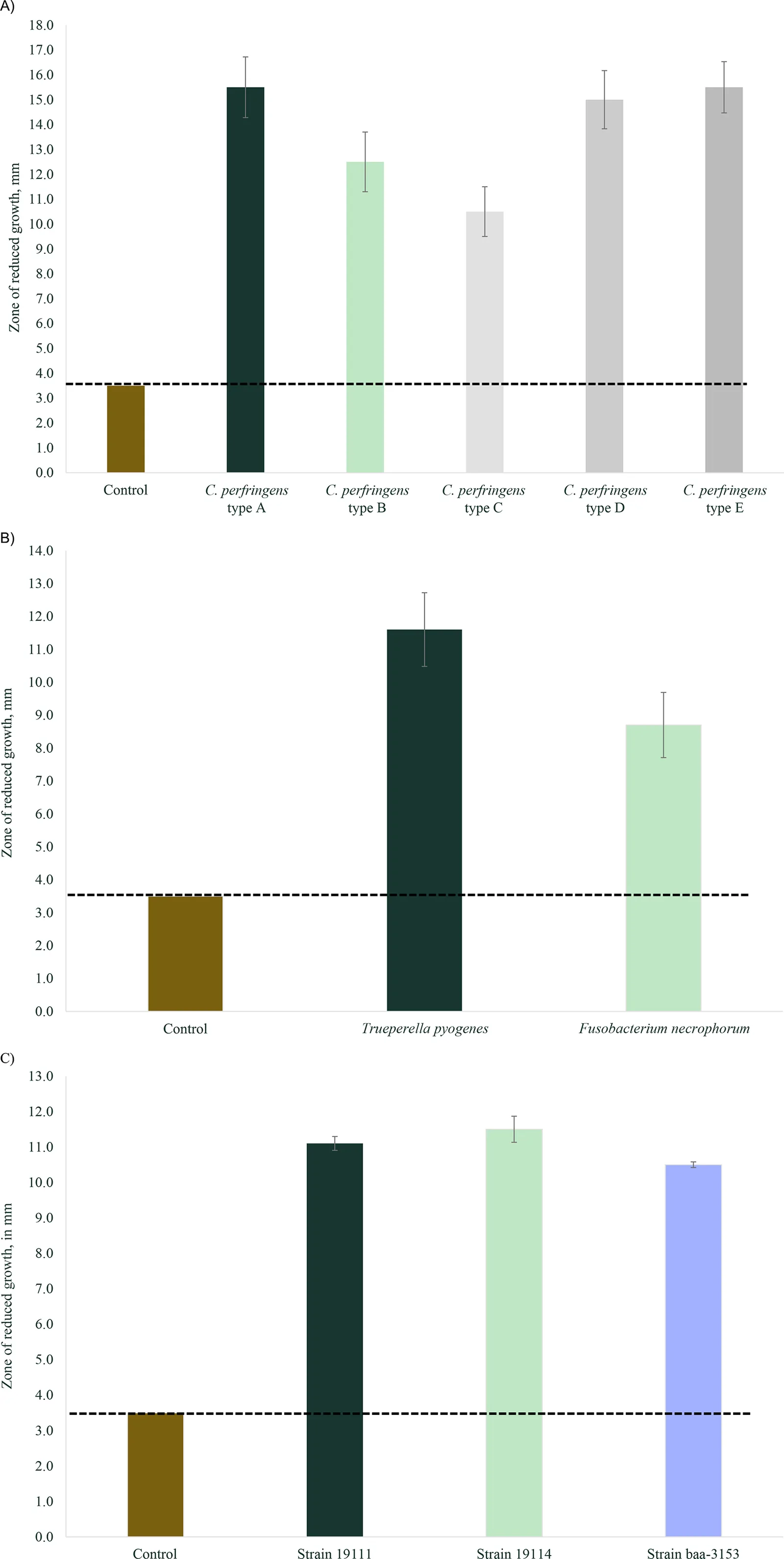
Zones of reduced growth (mm) of *Bacillus licheniformis* 809 and *B. subtilis* 810 when incubated under an in vitro agar-diffusion assay with *Clostridium perfringens* types A, B, C, D, and E (A), with *Fusobacterium necrophorum* and *Trueperella pyogenes* for 24 h (B), or with 3 strains of *Listeria monocytogenes* for 4 and 24 h (C). For all graphs, the dashed line represents the threshold for measurement (3.5 mm). Error bars are SEM.

The development of in vitro techniques to elucidate the distinct modes of action of bacteria-based DFM and how these could support the health and performance results from in vivo animal studies are paramount for a better understanding of the technologies available to the livestock industry. Hence, the goal of the current manuscript was to understand the effects of adding a *Bacillus*-based DFM containing BL and BS on the gas production and growth of *C. perfringens* type A, as well as on the efficacy against different potentially harmful bacteria of interest for dairy calves and cows, including *C. perfringens* types A thorough E, *F. necrophorum*, *T. pyogenes*, and *L. monocytogenes*. Last, it is important to recognize and emphasize that these are in vitro assays, and the goal was not to create a definite extrapolation of these results to live animals, but to provide a foundation for understanding why the health-supportive effects of BL and BS have been observed in dairy animals.

Enterotoxaemia caused by *C. perfringens* presents a mortality rate of roughly 100% and occurs when the growth of this pathogen and the production of toxins in the gastrointestinal tract are uncontrolled (Shrestha et al., 2018). In turn, these toxins may damage the intestinal epithelial cells and induce mucosal shedding and dysbiosis (Uzal et al., 2010). *Clostridium perfringens* types A, D, and B were the most common bacteria serotypes found in mature cattle and calves showing enterotoxaemia (Sasani et al., 2013). Moreover, vaccination against the different serotypes of *C. perfringens* does not provide full-herd protection (Nekrasov et al., 2023). We demonstrated that incubating BL or BS with *C. perfringens* type A significantly reduced gas production (up to 20%) over 24 h, whereas the counts of *C. perfringens* were reduced by up to 69%. The high level of in vitro gas production of *C. perfringens* is linked to a fast fermentation of carbohydrates and thus to a fast growth rate. The inhibition of in vitro gas production by *Bacillus* spp. could be the consequence of the inhibition of *C. perfringens* growth or a more efficient competition by medium nutrients. Even though a direct link between the inhibition of gas production and the inhibition of virulence cannot be stated, our results seem to indicate that *Bacillus* spp. can be competitive in terms of growth over *C. perfringens* in a closed system. It should be mentioned that pathogenicity was not properly evaluated herein to draw any further conclusions. In experiment 2, zones of reduced growth greater than 10.5 mm were observed in all *C. perfringens* serotypes, suggesting a positive effect of *Bacillus* spp. when co-incubated with these different serotypes. This in vitro series of studies does not provide definite and accurate answers on the modes of action of bacilli against *C. perfringens*, but complements previous work and reviews (Capern et al., 2025; Cappellozza et al., 2025). In beef cattle challenged with *C. perfringens* type A, supplementation of a multispecies bacteria-based DFM containing *B. licheniformis* 809 and *B. subtilis* 597 reduced the proportion of calves with adverse gastrointestinal events (Guimaraes et al., 2023). The occurrence of adverse gastrointestinal events was also reduced in crossbred preweaning calves fed BL and BS via whole milk (Magalhães et al., 2024).

*Fusobacterium necrophorum* and *T. pyogenes* are associated with the occurrence of uterine diseases in dairy cows (Bicalho et al., 2012), rumen challenges, and liver abscesses in feedlot cattle (Amachawadi and Nagaraja, 2016). Under a metagenomic analysis of the intrauterine microbial diversity, *F. necrophorum* was the most prevalent bacterium in the intrauterine environment of metritic cows, but absent in healthy cohorts (Santos et al., 2011). The frequent association of *T. pyogenes* with *F. necrophorum* in different pathologies is because of the nutritional and pathogenic synergy between them (Tadepalli et al., 2009). In dairy cows, their establishment in the uterine environment was preceded by *E. coli* (Bicalho et al., 2012), highlighting the codependence of the different bacteria in the different tissues of ruminants. Moreover, 90% of the dairy cows positive for a virulence factor (*fimH*) immediately postpartum were contaminated with *F. necrophorum* one week later, whereas its presence was recognized as an important risk factor for the appearance of *T. pyogenes* (Bicalho et al., 2012). Both bacteria were strongly associated with clinical endometritis at ∼35 DIM (Bicalho et al., 2012). Hence, our results demonstrating that BL and BS were effective when co-incubated with both *F. necrophorum* (8.5 mm) and *T. pyogenes* (11.5 mm) are an important initial validation on how *Bacillus*-based DFM may be able to modulate the microbial community of both gram-negative and gram-positive bacteria, respectively. Hence, all results presented here are in vitro, and additional studies are warranted to understand how a coculture of potentially harmful bacteria and DFM could influence the expression of virulence factors associated with the presence of potentially harmful bacteria.

However, it is not only in terms of reproductive function that both bacteria are important and relevant for livestock production. The ruminal concentration of *F. necrophorum* is often low (<10^6^) but is greater in cattle fed grain-based diets (Tan et al., 1994), likely due to the increased lactate formation and availability originated from the high-grain diet. Therefore, conditions that lead to lactate accumulation, such as subclinical and clinical acidosis, should promote the proliferation of *F. necrophorum*. The source of *T. pyogenes* in liver abscesses appears to be the ruminal wall and is more frequently isolated from the ruminal wall versus rumen contents (Narayanan et al., 1998). Because *T. pyogenes* is a facultative anaerobe, its niche is more likely to be the ruminal wall where oxygen is more available from the blood circulation in an otherwise anaerobic environment of the rumen. In a feedlot study, the supplementation of BL and BS to heat-stressed cattle reduced the incidence of liver abscess (19.0% vs. 7.3%; Mackey et al., 2024), suggesting a positive effect of the DFM on the rumen environment and in the balance of the microbial community.

*Listeria monocytogenes* is an important foodborne pathogen that is present in almost every environment, including edible products (McLauchlin et al., 2004; Bandelj et al., 2018; Chow et al., 2021). Hence, many countries have been taking actions to remove *Listeria* from the food chain, ensuring safer edible products (Rajabi et al., 2020). In dairy farms, season of the year (spring and summer) increased the risk of *L. monocytogenes* fecal shedding (Bandelj et al., 2018). Hence, given the ubiquitous presence of *Listeria*, one may speculate that ruminants may ingest *L. monocytogenes*, and strategies that alleviate the growth and presence of this bacterium might be required. Following oral ingestion, *L. monocytogenes* can cross the intestinal epithelium and translocate through epithelial cell junctions (Bonazzi et al., 2009; Drolia and Bhunia, 2019). In the gut lumen of mice, *L. monocytogenes* negatively affected the commensal microbial community through the production of bacteriocins (Quereda et al., 2016), whereas limited information has been reported for cattle (Chow et al., 2021). Overall, incubating BL and BS was efficacious against the different strains of *L. monocytogenes*, yielding clearance zones that ranged from 10.5 to 11.5 mm. Counts and virulence genes expression of *L. monocytogenes* were reduced following the co-incubation of *B. megaterium* and *B. laterosporus* (Rajabi et al., 2020). Although the exact mechanisms yielding such results are unknown, these might involve the production of compounds that are effective against this specific potentially harmful pathogen rather than a drop in pH.

In summary, these in vitro experiments demonstrated the supportive effects of BL and BS when exposed to *C. perfringens*, *F. necrophorum*, *T. pyogenes*, and *L. monocytogenes*. Nonetheless, it is important to mention that these studies were performed with pure cultures of both the probiotics and potentially harmful bacteria, and caution should be taken when exploring the current results. Additional studies are warranted to understand the in vivo health-supportive, immune, and performance responses of this specific *Bacillus*-based DFM in livestock exposed to potentially harmful bacteria.
